# Tachycardia in a teenager

**DOI:** 10.1007/s12471-021-01611-5

**Published:** 2021-08-03

**Authors:** Y. R. Persia-Paulino, J. Rozado, D. Perez

**Affiliations:** 1 Cardiology Department, Central University Hospital of Asturias, Oviedo, Spain; 2 Electrophysiology Department, Central University Hospital of Asturias, Oviedo, Spain

## Answer

**Fig. 1:**
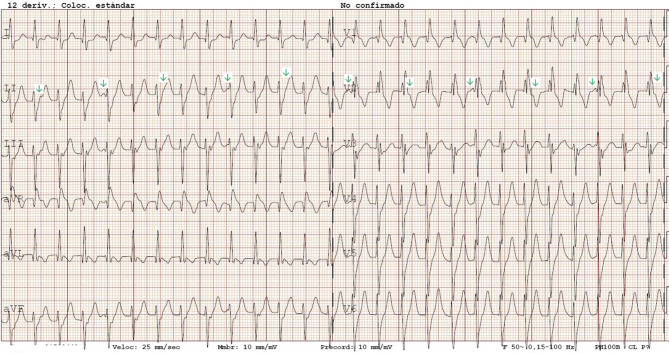
The 12-lead electrocardiogram on admission, with *green arrows* indicating P waves. Auricular and ventricular activity are dissociated (as P waves are not related in rhythm or frequency to QRS complexes)

The electrocardiogram on admission shows a regular tachycardia of approximately 162 beats/min with a relatively narrow QRS complex (116 ms) with a right bundle branch block (RBBB) morphology and left axis deviation (LAD). Atrioventricular dissociation can be seen (Fig. 1; green arrow shows P waves, which are not related in rhythm or frequency to QRS complexes). These findings are consistent with an idiopathic left ventricular tachycardia (ILVT) originating in the left posterior fascicle (which explains the RBBB and LAD in such a young patient). It is a re-entry tachycardia involving the interventricular septum, the apex and one of the left fascicles of the bundle of His (in this case, the left posterior fascicle, which is involved most frequently) [1].

ILVT is usually seen in patients aged 15–40 years with structurally normal hearts and has a low risk of sudden death (although deaths have been reported). Episodes usually occur at rest, but are influenced by exercise and emotional stress. Verapamil is usually effective in ending ILVT and preventing new episodes. Radiofrequency catheter ablation (RFCA) is an option for patients intolerant to antiarrhythmic drugs or with severe symptoms [2]. RFCA of ILVT has an overall success rate of 95% [3], but changes in surface ECG can occur, specifically the development of a Q wave on inferior leads and, less frequently, a left posterior fascicular block (as this fascicle, which is part of the tachycardia circuit, could be damaged) in approximately 10% of patients [4].

In wide QRS complex tachycardia, atrioventricular (AV) dissociation is a criterion that has been reported to have a 100% positive predictive value [5]. For differential diagnosis between supraventricular and ventricular tachycardia, the Brugada algorithm is very useful, as in this case AV dissociation is the only criterion met.
